# Salicylic acid in *Populus tomentosa* is a remote signalling molecule induced by *Botryosphaeria dothidea* infection

**DOI:** 10.1038/s41598-018-32204-9

**Published:** 2018-09-19

**Authors:** Yong-xia Li, Wei Zhang, Hui-xia Dong, Zhen-yu Liu, Jian Ma, Xing-yao Zhang

**Affiliations:** 1 Lab. of Forest Pathogen Integrated Biology, Research Institute of Forestry New Technology, Chinese Academy of Forestry, Beijing, 100091 China; 2 Co-Innovation Center for Sustainable Forestry in Southern China, Nanjing Forestry University, Nanjing, 210037 China; 3 College of Life Science, Henan Normal University, Xinxiang, 453007 China; 4 College of Plant Protection, Shandong Agricultural University, Tai’an, 271018 China

## Abstract

The salicylic acid (SA) plays a critical role during the establishment of systemic acquired resistance (SAR) in uninfected plant tissues after localised exposure to a pathogen. Here, we studied SA in *Populus tomentosa* infected by the plant pathogen *Botryosphaeria dothidea*. The accumulation of SA and methyl salicylate (MeSA) occurred in chronological order in *P*. *tomentosa*. The SA and MeSA contents were greater at infected than uninfected sites. Additionally, a gene expression analysis indicated that SA might be accumulated by phenylalanine ammonialyase (PAL) and converted to MeSA by SA carboxyl methyltransferase (SAMT), while MeSA might convert to SA by SA-binding protein 2 (SABP2). The expressions of *SAMT* at infected sites and *SABP2* at uninfected sites, respectively, were significantly up-regulated. Thus, SA might be converted to MeSA at infected sites and transported as a signalling molecule to uninfected sites, where it is converted to SA for SAR. Moreover, the expressions of pathogenesis-related genes *PR-1*, *PR-2* and *PR-5* in *P*. *tomentosa* were up-regulated by the *B*. *dothidea* infection. Our study determined that variations in SA and MeSA contents occur at infected and uninfected sites in poplar after pathogen infection and contributed to the remote signals for poplar SAR.

## Introduction

During evolution, plants evolved complex mechanisms to adapt to pathogen attack, wounds, heat and other environmental stresses^1–4^. These mechanisms include the rapid increase of signalling molecules, such as salicylic acid (SA), jasmonic acid and ethylene^5^. In recent years, SA has generally been considered a critical signal transduction factor in the defence against biotrophic pathogens^6^. Phenylalanine ammonialyase (PAL) is a key enzyme in the SA biosynthetic pathway^7,8^. The accumulation of endogenous SA in the healthy systemic tissue of infected plants is required for the expression of many defence genes, including pathogenesis-related (*PR*) genes, and the activation of systemic acquired resistance (SAR)^9–11^. SA-dependent SAR is a long-lasting, broad-spectrum resistance that is established in uninfected parts of plants after localised pathogen exposure^12–14^. However, SA itself is most probably not the mobile signal inducing SAR based on grafting experiments using transgenic tobacco expressing the SA-degrading salicylate hydroxylase encoded by *Nah*G as rootstocks for primary infections^15^. In fact, methyl salicylate (MeSA), a derivative of SA, plays a pivotal role as a long-distance mobile signal for SAR in uninfected parts of plants^16,17^. MeSA is formed from SA by SA carboxyl methyltransferase (SAMT), and converted back to SA by SA-binding protein 2 (SABP2)^18^. SABP2’s enzymatic activity is required in systemic tissues to mount a successful SAR response to pathogen infection^19^. The reduction of MeSA in primary infected leaves by either the overexpression of a mutant SABP2 or the silencing of SAMT results in the loss of SAR and the suppression of *PR* gene expression levels^20,21^. Thus, MeSA is an essential part of the SAR signal in plants and it can coexist with SA, transmuting into each other, as needed, through the activities of SAMT and SABP2.

Thus, it is important to study SA as a universal signalling molecule in all plants. SA accumulation could induce SAR in relatively sensitive plants, such as tobacco (*Nicotiana tabacum*), cucumber (*Cucumis sativus*) and Arabidopsis (*Arabidopsis thaliana*). However, some plants, like rice (*Oryza sativa*) and potato (*Solanum tuberosum*), contain very high basal SA levels, which make it difficult to act as an effective signalling molecule to activate many defence genes and induce disease resistance^22,23^. Thus, SA’s functional mechanisms during immune responses in different plant–pathogen interaction systems may be completely different. The roles of the SA-dependent signalling pathway in a variety of annual herbs, such as *Arabidopsis*^24,25^, tobacco^13^ and rice^26^, have been well studied. However, very little is known about the role of the SA signalling pathway in response to pathogen challenge in perennial woody species, which have longer life spans and are more likely to experience pathogen attacks^27^.

Poplar (*Populus* species) is a widespread and economically important wood resource worldwide^28^. *Botryosphaeria dothidea* (Mougeot ex Fries) Cesati et de Notaris is a plant pathogen that causes the formation of cankers on a wide variety of tree and shrub species. This species infects several hundred plant hosts on all continents, except Antarctica^29,30^. In north-central China, *B*. *dothidea* causes stem cankers on poplar. It can reduce the growth of plants or even kill them, resulting in significant production losses, as large as 70%, for a plantation^31^. The development of a *Populus* species as a model for forest trees provides an unprecedented opportunity to expand our understanding of the interactions between fungi and woody perennial plants. SA treatments can induce the expression of defence genes in poplar^32^, suggesting that the SA signalling pathway may be very important for SAR in poplar.

Despite the importance of SA and MeSA in defence signalling in herbaceous plants, whether they play roles in poplar is unclear. There are limited reports on the effects of SA and MeSA on poplar stem canker^33,34^. To investigate the function of SA in poplar interactions with *B*. *dothidea*, we monitored SA and MeSA levels in infected and uninfected sites on *P*. *tomentosa* inoculated with *B*. *dothidea*, cloned *SAMT* and *SABP2* from *P*. *tomentosa*, measured the expression levels of *SAMT*, *SABP2* and *PR* genes, and analysed the relationships among SA, MeSA, SAMT and SABP2. Our aim was to clarify the role of SA and its signal transduction mechanism during *P*. *tomentosa–B*. *dothidea* interactions.

## Results

### SA and MeSA accumulated in *P*. *tomentosa* after *B*. *dothidea* inoculation

To investigate the functions SA in *P*. *tomentosa* infected with *B*. *dothidea*, we determined the SA and MeSA levels in *P*. *tomentosa* at 0, 6, 12, 24, 48, 72 and 96 h after inoculation with *B*. *dothidea*.

**Figure 1:**
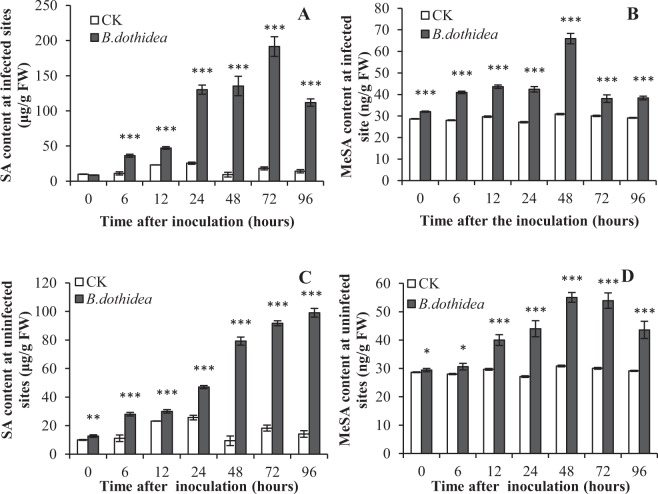
Liquid chromatography of SA in P. tomentosa after B. dothidea inoculation. SA, Free salicylic acid. MeSA, methyl salicylate. Infected sites, 0.5 cm around inoculation sites. Uninfected sites, 10–15 cm away from inoculation sites. □, CK (control) inoculation with culture-medium; ■, inoculation with *B*. *dothidea*. Error bars represent SEM. P values for differences between inoculation with *B*. *dothidea* and control: *P < 0.05, **P < 0.01 and ***P < 0.001 (Independent-samples T test).

The SA content in *P*. *tomentosa* increased significantly near the infection sites 6 h after inoculation with *B*. *dothidea* and continued to accumulate to the peak 191.42 µg/g fresh weight (FW) by 72 h, after which the value gradually decreased (Fig. 1A). The SA levels in the control remained low (from 9.98 to 14.12 µg/g FW) throughout the sampling period. MeSA is a derivative of the SA, and they can transmute between forms. To further define the role of SA and its correlation to the MeSA content, we determined the MeSA content, which was significantly increased in *P*. *tomentosa* after inoculation with *B*. *dothidea*. The controls showed no significant changes (ranging from 28.65 to 29.13 ng/g FW). As the time after inoculation with *B*. *dothidea* increased, the MeSA content increased steadily from 6 h to 48 h, peaked at 65.93 ng/g FW at 48 h, and then decreased from 72 h to 96 h (Fig. 1B). After *B*. *dothidea* inoculation, the SA contents increased rapidly (8.67 ng/g FW at 0 h to 191.42 ng/g FW at 72 h). However, the MeSA content increased slowly (from 32 ng/g FW at 0 h to 66 ng/g FW at 48 h). In addition, the peak time for MeSA occurred at 48 h, which was earlier than that of SA at 72 h.

In addition to the SA and MeSA contents at infected sites, we measured their contents at uninfected sites far from the *B*. *dothidea*-inoculation point in the same poplars. They showed trends that were similar to those at the infected sites. In uninfected tissues, both the SA and MeSA contents were higher than in the controls. Compared with the MeSA contents, the SA contents increased rapidly. As the time after inoculation increased, the SA contents in poplar increased gradually to 99.01 ng/g FW at 96 h (Fig. 1C). The MeSA contents in poplar also increased from 6 h to 48 h, peaked at 55.04 ng/g FW at 48 h, and then decreased from 72 h to 96 h (Fig. 1D).

Thus, SA and MeSA contents increased after inoculation with *B*. *dothidea* in both the infected and uninfected sites.

### Expression analyses of *SAMT* and *SABP2* in *P*. *tomentosa* after inoculation with *B*. *dothidea*

SAMT and SABP2 are two key enzymes in the reciprocal transmutation between SA and MeSA. SA is converted to MeSA by SAMT, and SABP2 mediates the hydrolysis of MeSA to SA. To further determine whether a conversion exists between SA and MeSA, and to clarify their roles as regulatory factors in poplar, we cloned the *SAMT* and *SABP2* genes. *P*. *tomentosa* first-strand cDNA was used as a template, and the *SAMT* and *SABP2* genes were amplified with specific primers and checked by 1.5% agarose gel electrophoresis. Then, the fragments were recovered and sequenced by the Nomad Biological Company. The full-length coding sequence of PtoSAMT (GenBank No. JQ086572) was 1,092 bp, encoding a 364-amino acid protein. The full-length coding sequence of SABP2 was 787 bp, and PtoSABP2 (GenBank No. JQ086570) encoded a 262-amino acid protein.

**Figure 2:**
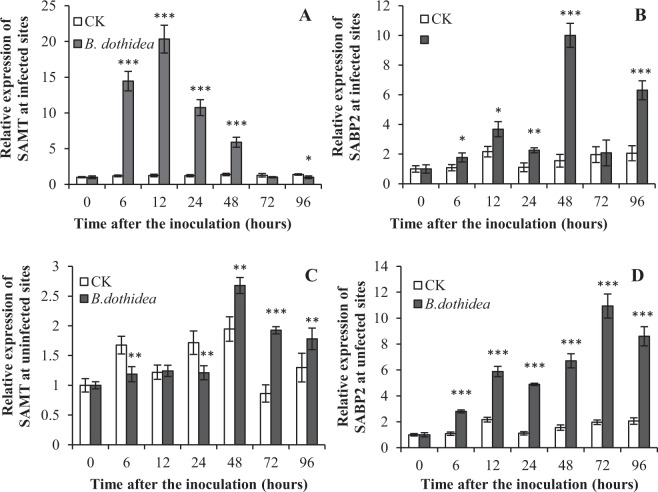
Expression of SAMT and SABP2 in P. tomentosa after inoculation with B. dothidea. SAMT, salicylic acid methyltransferases. SABP2, salicylic acid binding protein 2. Infected sites, 0.5 cm around inoculation sites. Uninfected sites, 10–15 cm away from inoculation sites. □, CK (control) inoculation with culture-medium; ■, inoculation with *B*. *dothidea*. Error bars represent SEM. P values for differences between inoculation with *B*. *dothidea* and control: *P < 0.05, **P < 0.01 and ***P < 0.001 (Independent-samples T test).

To determine the expression kinetics of the *SAMT* and *SABP2* genes of *P*. *tomentosa* at different times after inoculation with *B*. *dothidea*, we measured the expression levels of the *SAMT* and *SABP2* genes at infected sites. Both *SAMT* and *SABP2* fluctuated significantly in *P*. *tomentosa* after inoculation with *B*. *dothidea*, while both *SAMT* and *SABP2* showed lower expression levels and relatively smooth changes in the control after inoculation with culture medium (Fig. 2A,B). Moreover, the change patterns were strikingly different between *SAMT* and *SABP2*. The expression of *SAMT* increased and peaked at 12 h, decreased significantly by 72 h after inoculation, and reached its lowest level at 96 h after inoculation. In contrast, the expression of *SABP2* increased rapidly, peaked at 48 h after infection, decreased by 72 h, and increased again at 96 h. *SABP2*’s peak expression time was later than that of *SAMT*. Importantly, the *SAMT* and *SABP2* expression changes occurred prior to the changes in SA and MeSA contents, respectively.

The expression levels of *SAMT* and *SABP2* genes were also measured at uninfected sites. *SABP2* gene expression was up-regulated in uninfected tissues after inoculation with *B*. *dothidea* compared with that of the control at almost all tested times (Fig. 2C,D). The expression level began to rise 6 h after infection, and the highest value occurred at 72 h. The *SAMT* gene’s expression level initially seemed un- or down-regulated compared with the control. Then, after 48 h, it was up-regulated. However, the range of the changes was smaller than that of *SABP2*. The *SAMT* gene’s expression in uninfected tissues was low relative to its level in infected tissues.

Thus, at infected sites, the expression level of *SAMT* was up-regulated earlier and to a greater extent, while the peak time of *SABP2* occurred later than that of *SAMT*. However, at uninfected sites, *SABP2* was up-regulated earlier, while the SAMT level initially remained low.

### Up-regulation of PAL in SA synthesis

SA increased rapidly after inoculation with *B*. *dothidea* at both infected and uninfected sites. PAL activity leads to the accumulation of SA in plants (Pan *et al*., 2006). Therefore, we determined the expression and enzyme activity levels of PAL at infected sites.

**Figure 3:**
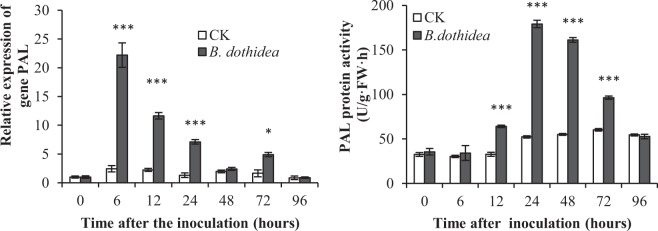
Expression and protein activity of PAL in P. tomentosa after inoculation with B. dothidea. PAL, phenylalanine ammonia. □, CK (control) inoculation with culture-medium; ■, inoculation with *B*. *dothidea*. Infected sites were 5 mm around the inoculation sites and uninfected sites were 10 cm away from inoculation sites. Error bars represent SEM. P values for differences between inoculation with *B*. *dothidea* and control: *P < 0.05, **P < 0.01 and ***P < 0.001 (Independent-samples T test).

The expression of *PAL* was significantly up-regulated in *P*. *tomentosa* 6 h after *B*. *dothidea* inoculation and decreased significantly by 48 h after inoculation (Fig. 3). In the control, there was no significant change after inoculation. Thus, the expression of the *PAL* gene in *P*. *tomentosa* was affected by the *B*. *dothidea* infection and possibly caused the accumulation of SA (Fig. 1).

Moreover, the activity levels showed a similar variation trend as the expression in Poplar after inoculation with *B*. *dothidea*. PAL activity peak at 24 h after inoculation (up-regulated 3.4-fold) (P < 0.001), decreased gradually again from 72 h to 96 h after inoculation, and eventually returned to the control level (Fig. 3).

### Defence-related gene expression levels in *P*. *tomentosa* after inoculation with *B*. *dothidea*

**Figure 4:**
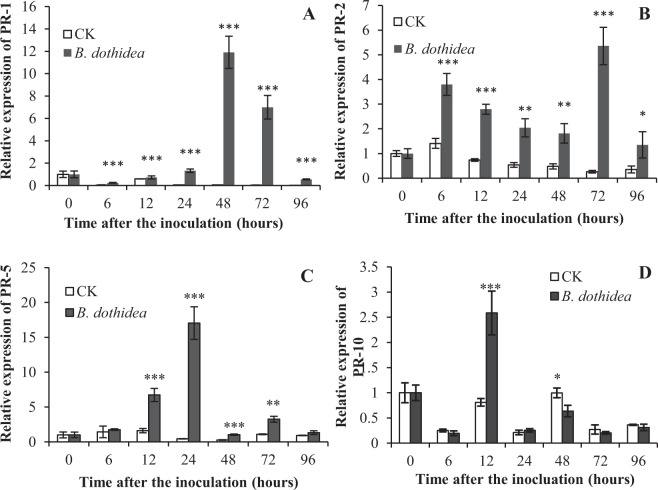
Expression of PR genes in P. tomentosa after inoculation with B. dothidea. PR genes, pathogenesis related genes. □, CK (control) inoculation with culture-medium; ■, inoculation with *B*. *dothidea*. Infected sites were 5 mm around the inoculation sites and uninfected sites were 10 cm away from inoculation sites. Error bars represent SEM. P values for differences between inoculation with *B*. *dothidea* and control: *P < 0.05, **P < 0.01 and ***P < 0.001 (Independent-samples T test).

Commonly, during pathogen attacks, SA signalling molecules induce the expression of *PR* genes for plant defence. To study variations in *PR* gene expression in *P*. *tomentosa* after inoculation with *B*. *dothidea*, the expression levels of four defence-related genes (*PR-1*, *PR-2*, *PR-5* and *PR-10*) were measured at infected sites of poplar. Following the stem inoculation with *B*. *dothidea*, the *PR-1* expression level in *P*. *tomentosa* was not elevated prior to 48 h but it significantly increased at 48 h compared with the control and decreased significantly by 96 h (Fig. 4A). The level of *PR-2* expression was much greater at 6 h and peaked at 72 h (Fig. 4 B), while that of *PR-5* peaked at 24 h (Fig. 4C). However, for *PR-10*, the expression was up-regulated at 12 h, while the other time points were almost stable. In the control, there was no significant change in the expression levels of the four genes after pathogen inoculation. Thus, the expression levels of *PR-1*, *PR-2* and *PR-5* in *P*. *tomentosa* were affected by the *B*. *dothidea* infection.

## Discussion

SA is necessary for activating plant resistance and plays an important role in plant defence mechanisms^35–37^. Free SA and SA glucoside (SAG, a storage form of SA) are two forms of SA in plants. Large amounts of SA accumulated in *P*. *tomentosa* tissues after inoculation with *B*. *dothidea*, while the control did not induce SA accumulation under the same conditions, indicating that the changes in SA were closely correlated with the *B*. *dothidea* infection. This was consistent with plants accumulating large amounts of SA to activate defence mechanisms under stress conditions^1^. The SAG content increased rather than decreased (Fig. S2), which indicated that free SA played a key role in the interaction between the *P*. *tomentosa* and *B*. *dothidea* and that SA was synthesised by other pathways. Thus, in addition to SA from SAG, in plants, increases in SA are derived from the biosynthetic pathway in which PAL is the key enzyme^7^. In our study, the SA content increased according as the PAL enzymes activity and expression levels were up-regulated. The accumulation of endogenous SA in infected plants is essential for *PR* gene expression^9–11^, and this was corroborated by the up-regulated expression levels of the four *PR* genes in *P*. *tomentosa* after infection with *B*. *dothidea*.

Plants accumulate a large amount of SA to activate the defence reaction mechanisms under stress conditions^12^, and some of the SA can be converted into MeSA near the site of infection. MeSA then acts as a signalling molecule to communicate with uninfected plant parts. MeSA is transformed into SA again by the SABP2 protein at uninfected sites, after which the plant defence mechanisms are activated by SA^38^. Our study demonstrated that large amounts of SA and MeSA accumulated in *P*. *tomentosa* tissues after inoculation with *B*. *dothidea*, indicating that the changes in SA and MeSA contents were closely correlated with the *B*. *dothidea* infection (Fig. 1). Importantly, our data indicated a chronological order for the accumulation of the SA and MeSA contents in *P*. *tomentosa* tissues after inoculation with *B*. *dothidea*. The SA levels were slightly higher in *P*. *tomentosa* at 6 h after inoculation with *B*. *dothidea*, and increased gradually between 12 h and 72 h, while the MeSA levels increased at 48 h after inoculation. Thus, there might be a conversion from SA to MeSA. After 48 h, the MeSA levels decreased rapidly, while the SA content continued to increase, suggesting that MeSA might have been converted back to SA. Thus, MeSA might act as a signalling molecule and play a key role in the induced defence reaction of SA.

In plants, SA is synthesised through two routes. One route involves isochorismate synthase, which is believed to be responsible for >90% of SA synthesised during the activation of stress responses. The other route uses the PAL-mediated pathway^18^. PAL is also essential for SA accumulation and SAR^39,40^. To date, the contributions of the two synthesis pathways to SA levels were not clear^41^. In our studies, the expression and enzyme activity of PAL were significantly up-regulated in *P*. *tomentosa* 6 h after *B*. *dothidea* inoculation and decreased significantly 48 h after inoculation (Fig. 3). Thus, the expression level of the *PAL* gene in *P*. *tomentosa* was affected by the *B*. *dothidea* infection and related to the SA accumulation (Fig. 1).

SAMT and SABP2 are required for SAR^18,42^. A large amount of SA accumulated at the site of infection and activated defence responses in the plant. Some of the SA can be converted into MeSA catalysed by SAMT, and MeSA is transported to the uninfected site through trachea, and it re-forms SA catalysed by SABP2 to activate the defence responses of uninfected plant tissues^19^. Our results showed the same timeline. At infected sites the expression levels of *SAMT* were up-regulated earlier and to a greater extant, which might contribute to MeSA synthesis for long-distance transportation. The peak time of *SABP2* expression was later than that of *SAMT*. However, at uninfected sites, *SABP2* was up-regulated first, which might elevate SA levels through the hydrolysis of MeSA, while SAMT levels initially remained low. Thus, we hypothesise that SA and MeSA had important roles in the signal transduction necessary for the defence response of poplar against pathogen attack. The conversion between MeSA and SA might be regulated by SAMT and SABP2 in both infected and uninfected tissues^12,19^. Our findings suggested that SABP2 mediates SA signalling through the demethylation of MeSA and that the reverse reaction (SA to MeSA catalysed by SAMT) was also necessary for disease defence in poplar. These results were consistent with previous observations that SA and MeSA were crucial signals for the activation of defence responses, suggesting that SAMT and SABP2 might be induced for proper signalling in poplar.

The regulation of *PR* gene expression is a biological effect and a sign of SAR^43^. In particular, *PR-1*, *PR-2* and *PR-5* are markers of SAR^44,45^. An increased SA content could induce the expression of *PR* genes in plants after pathogen infection^46–48^. Here, the expression levels of the defence-related genes *PR-1*, *PR-2* and *PR-5* were significantly up-regulated in *P*. *tomentosa* after inoculation, which indicated that *P*. *tomentosa* initiated the SA signalling pathways upon *B*. *dothidea* infection. Thus, poplar may activate the SA signalling pathway as a remote signal for SAR and the defence reactions after *B*. *dothidea* infection.

## Methods

### Materials, inoculations and samples

One-year-old plants of *P*. *tomentosa* (tolerant to *B*. *dothidea;* Disease Index of Canker in nature: 0–6.0) were cultured in pots (24-cm diameter) containing compost in a greenhouse with a day/night cycle of 10 h at 25 °C/14 h at 20 °C, with a constant relative humidity of 70%.

The *B*. *dothidea* used in this study was isolated from the canker lesions of *P*. × *beijingensis* in the Haidian District of Beijing, China and was preserved in the Forest Pathology Laboratory of the Research Institute of Forest Ecology, Environment and Protection, Beijing, China. Before inoculation, *B*. *dothidea* was activated on potato dextrose agar for 6 d at 25 °C in the dark. For inoculation, 5-mm wounds were created in the bark of each stem with a dissecting needle. Then, 5-mm plugs of potato dextrose agar without (control) or with *B*. *dothidea* were placed in the wound, packed in with sterile cotton and sterile water, and sealed with plastic wrap to retain moisture.

After inoculation, samples were taken at 0, 6, 12, 24, 48, 72 and 96 h at the infected sites (5 mm around the inoculation site) and uninfected sites (10 cm away from the inoculation site). The samples (containing cambiums) were immediately frozen in liquid nitrogen and stored at −80 °C for biochemical analysis, and 10 samples from each tree were mixed in equal volumes and used as one sample. Each treatment had three replicates (three trees).

### Analysis of SA and MeSA contents in *P*. *tomentosa* after inoculation with *B*. *dothidea*

The SA extraction was based on a previous method^49^. Phloem samples were collected and frozen in liquid nitrogen. Prior to extraction, all of the materials were pulverised in liquid nitrogen using a mortar and pestle. Samples of 1.0 g were further homogenised using liquid nitrogen and transferred to 1.5-ml Eppendorf tubes. An aliquot (1 ml) of 80% methanol was added to the homogenate, and the samples were centrifuged for 5 min at 10,000 g in a centrifuge. The supernatant was collected in a 2-ml Eppendorf tube, and the centrifugation steps were repeated. The supernatants were combined and placed in a −20 °C refrigerator for 1 h. The supernatants were divided into two parts, one for determining the free SA and the other the SAG contents. Then, 2 mM HCl was added to the each of the supernatants, mixed and moved to sealed tubes. They were incubated in an 80 °C bath for 1 h, to transform SAG to free SA, to measure total SA. Then, 2 ml of 5% trichloroacetic acid (TCA; 5% solution in water) was added to the supernatants, which were partitioned with 3 ml of ethyl acetate:cyclohexane (1:1, v/v). This partitioning was carried out three times. The combined upper layers were evaporated to dryness in a SpeedVac concentrator. The samples were dissolved in 1 ml of 70% methanol. The C18 column was washed with 5 ml of 70% methanol, the sample was passed through the column, and the column was eluted with the addition of 3 ml of 70% methanol. Then, the wash eluates were combined and evaporated to dryness in a SpeedVac concentrator. After removal from the concentrator, 250 μl of the HPLC eluent was added to each tube, and the SA contents were analysed by HPLC. The SA analysis was performed on an HPLC (Waters 2695, USA) column, sunfire C18 (4.6 mm × 250 mm, i.d.: 0.45 μm). The eluent was 70% methanol, pH 3, at a flow rate of 0.80 ml/min, retention time of 10.943 min, and injection volume of 20 μl.

The MeSA extraction protocol was based on a previous method^16^. Samples were flash-frozen in liquid nitrogen and stored at −80 °C until analysis. After grinding to a fine powder in liquid nitrogen, 1 g of phloem tissue (fresh weight) was extracted twice with 5 ml of 80% ethanol, placed in a −4 °C refrigerator overnight and centrifuged for 30 min at 12,000 rpm. The supernatant was collected and condensed in a vacuum. Then, 4 ml of ethyl acetate was added to the supernatant and mixed by vortexing until the ethyl acetate phase was colourless. The sample was generated by collecting the ethyl acetate phase and distilling to remove ethyl acetate. The sample was detected by gas chromatography, in which the chromatogram peaks were evaluated using their retention times. The injection port and detector temperatures were set to 250 °C and 270 °C, respectively. The initial oven temperature was held at 50 °C for 1 min and was programmed to increase at 10 °C per min to 200 °C, and held for 30 min, before cycling to the initial conditions. Each treatment had three replicates.

### PAL enzyme activity determinations in *P*. *tomentosa* after inoculation with *B*. *dothidea*

The PAL enzyme activity was determined using the methods described in The Modern Plant Physiology Experiment edited by the Chinese Academy of Sciences, Shanghai Institute of Plant Physiology^50^. Samples of 1.0 g from infected sites were ground to homogenates, then centrifuged for 20 min at 12,000 rpm at 4 °C. The supernatant fluid is required for the enzyme analysis. Then, 2 ml 0.1 M borate buffer (pH 8.8) and 1 ml 0.02 M L-phenylalanine were added to 1 ml enzyme liquid, and the OD value was immediately determined at 290 nm using an ultraviolet spectrophotometer. At a constant temperature of 37 °C, 5 M HCl was added to terminate the reaction after 1 h, and a second of OD_290_ value was determined. The enzyme activity unit is 0.01 of the OD change. Each treatment had three replicates.

### Identification of the *SAMT* and *SABP2* genes, which are putatively associated with resistance in *P*. *tomentosa*

The total RNA was isolated using an RNA Isolation Kit (BioTeke, Beijing, China) from stems that were or were not infected with *B*. *dothidea* according to the manufacturer’s instructions. The total RNA (2 µg) was reverse-transcribed into first-strand cDNA in a 20 µl reaction volume using a First-Strand cDNA Synthesis Kit (BioTeke). *Pto*SABP2 full-length cDNA was amplified from stem cDNA using the forward primer 5′-ATGGTAGAGACCAAGAATCAGGA-3′ and the reverse primer 5′-AGCATGTTTATTTGCTATCTCTGA-3′ from the sequences of the closely related species *Populus trichocarpa*. For *Pto*SAMT, the full-length cDNA was amplified from stem cDNA using the forward primer 5′-ATGGAGGTTGCTCAAGTGCTTCA-3′ and the reverse primer 5′-TCCCTTTCTAGTCACGGAAACAG-3′. The PCR proceeded as follows: 94 °C for 4 min, followed by 30 cycles of 94 °C for 30 s, 58 °C for 30 s and 72 °C for 1 min, and a final extension at 72 °C for 4 min. The PCR reaction was performed using hot start EX Taq polymerase (TaKaRa Bio, Japan), after which the product was ligated to the pEASY-T3 Cloning Vector and transformed into Trans1-T1 Phage Resistant Chemically Competent Cells (TransGen Biotech, Beijing, China). Clones were obtained and sequenced by Invitrogen. Phylogenetic trees of SAMT and SABP2 were constructed with homologous proteins from other species using MEGA^51^.

### The expression analysis of poplar defence-related genes using RT-qPCR

The total RNA extractions from the infected stems of one-year-old poplar trees that were treated with *B*. *dothidea* and subsequent first-strand cDNA synthesis were performed essentially as for full-length cDNA cloning. The sequences of the five poplar defence-related genes, *PR-1*, *-2*, -5 and *-10*, and *PAL*, were submitted to the NCBI database by other researchers, and the cDNA samples were analysed by RT-qPCR using gene-specific primers (Table S1), The endogenous β-tubulin gene was used as a control because its expression remains relatively constant. Phylogenetic trees for PR-1, PR-2, PR-5, PR-10 and PAL were constructed with homologous genes of other species using MEGA (Fig. S1).

RT-qPCR amplification was performed using the SYBR Premix EX Taq Kit (TaKaRa) in a 20 µl reaction volume, using 2 µl of 20-fold diluted cDNA as a template, 200 nM universal RT-qPCR primer and 200 nM miRNA-specific forward primers. The reactions were conducted on a 7500 Real-Time PCR System (Applied Biosystems, Foster City, CA, USA) using the TaKaRa-recommended cycling conditions. Each test had three experimental and two technical replicates.

### Statistical analyses

Excel 2007 was used to statistically analyse the SA and MeSA contents, as well as the expression levels of SABP2, SAMT, PR and PAL. All of the data are presented as the means ± SD, and were evaluated using the unpaired two-tailed Student’s t test or Mann-Whitney U test, depending on the results of the normality and homogeneity tests conducted using SPSS 13.0 software^52^.

## Electronic supplementary material

Supplemental data
