# Supplementary material for: Salicylic acid in Populus tomentosa is a remote signalling molecule induced by Botryosphaeria dothidea infection
Source: Sci Rep. 2018 Sep 19;8:14059. doi: 10.1038/s41598-018-32204-9 (PMC6145909; doi:10.1038/s41598-018-32204-9)
Supplement: Supplementary file 1 — Supplemental data [file 41598_2018_32204_MOESM1_ESM.pdf]

Salicylic acid in *Populus tomentosa* is a remote signalling molecule induced by the *Botryosphaeria dothidea* infection

Yong-xia Li[1, 2☯], Wei Zhang[1, 2☯], Hui-xia Dong[1, 3], Zhen-yu Liu[2, 4], Jian Ma[1, 2], Xing-yao Zhang[1, 2]

[1]Lab. of Forest Pathogen Integrated Biology, Research Institute of Forestry New Technology, Chinese Academy of Forestry, Beijing l00091, China

[2]Co-Innovation Center for Sustainable Forestry in Southern China, Nanjing Forestry University, Nanjing 210037, China

[3]College of Life Science, Henan Normal University, Xinxiang 453007, China

[4]College of Plant Protection, Shandong Agricultural University, Tai'an 271018, China

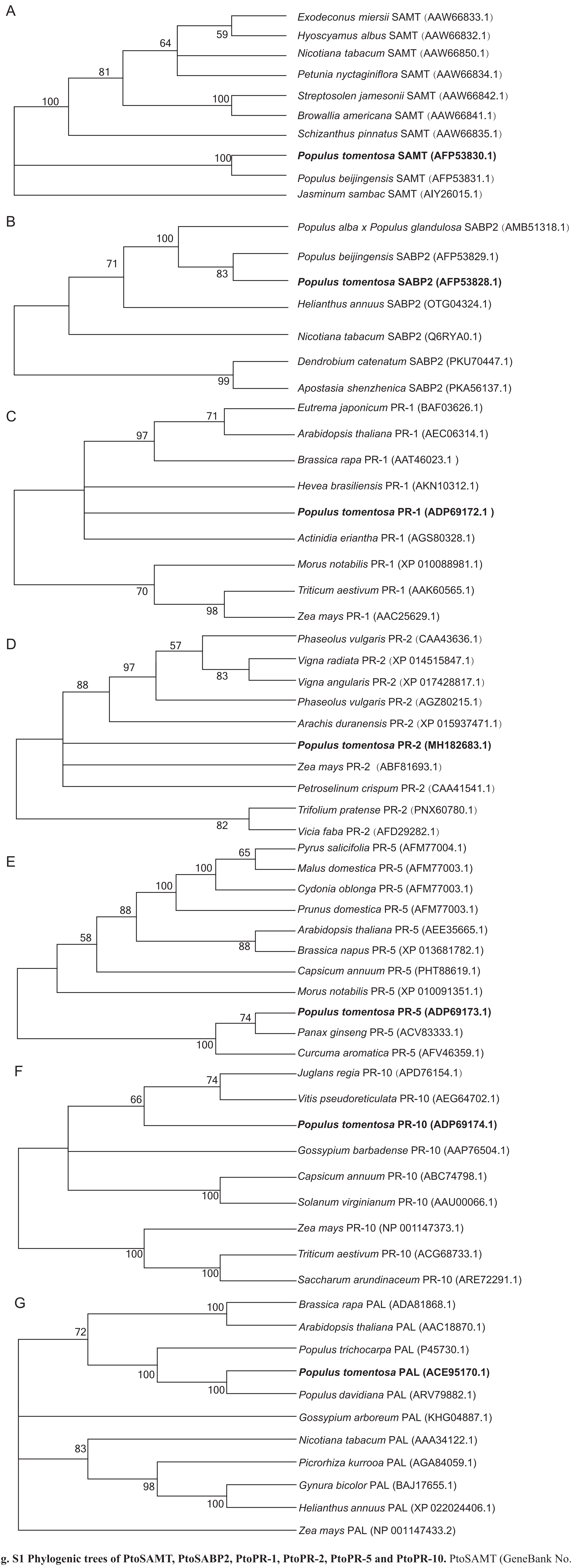

Fig. S1 Phylogenic trees of PtoSAMT, PtoSABP2, PtoPR-1, PtoPR-2, PtoPR-5 and PtoPR-10. PtoSAMT (GeneBank No. JQ086572) and PtoSABP2 (GeneBank No. JQ086570) sequences were submitted by us. Other sequences were from NCBI.

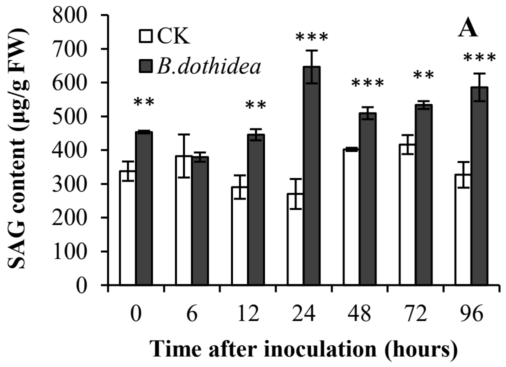

Fig. S2 Liquid chromatography of SAG in *P. tomentosa* after *B. dothidea* inoculation. SAG, SA glucoside (a storage form of SA). □, CK (control) inoculation with culture-medium; ■, inoculation with *B. dothidea.* Infected sites were 5 mm around the inoculation sites and uninfected sites were 10 cm away from inoculation sites. Error bars represent SEM. P values for differences between inoculation with *B. dothidea* and control: *P<0.05, **P<0.01 and ***P<0.001 (Independent-samples T test).

Table S1 Primer sequences for the qRT-PCR analysis of *SAMT*, *SABP2,* and PR genes in *P. tomentosa* following inoculation with *B. dothidea.*

Target gene	GenBank No.	Forward primer sequence (5'–3')	Reverse primer sequence (5'–3')
SABP2	JQ086570	GTTTGAGTTTGGCTCTTGCT	TGTGGTATCTGGCATGAAAG
SAMT	JQ086572	CAGCACAAGTCCACCAAGCGT	TTGAGAGCCACAGCCAAAAGC
PR-1	HM589199	GCTATAACAATCCCTCTATCCCTT	CCACACAATATTTCCAACACCTAC
PR-2	MH182683	TACCAATGACTGGGAGGAGCAC	AAGACACGGCGGACATAGAGC
PR-5	HM589200	ATCGTTGTTTCCCTGCTTCC	AAATTGCACTTGGTCCGTCC
PR-10	HM589201	ATGAGTTTTCTGTCGCTGTCCC	ACCTTCCTTATTGTTCCTGGCC
PAL	EU760386	TGAAGAAATAAGTCCCAACGAGT	TCAAAGGAGCTGAAATAGCAATG
β-Tubulin	MH173049	GCACCAACTTGTTGAGAATGC	TTTCAACTGACCAGGGAACC
